# Gustaw Bikeles (1861–1918)

**DOI:** 10.1007/s00415-022-11416-0

**Published:** 2022-10-07

**Authors:** Slawomir Gonkowski, Oksana Zayachkivska

**Affiliations:** 1 Department of Clinical Physiology. Faculty of Veterinary Medicine, University of Warmia and Mazury, Olsztyn, Poland; 2 Physiology Department, Danylo Halytsky Lviv National Medical University, Lviv, Ukraine

**Fig. 1:**
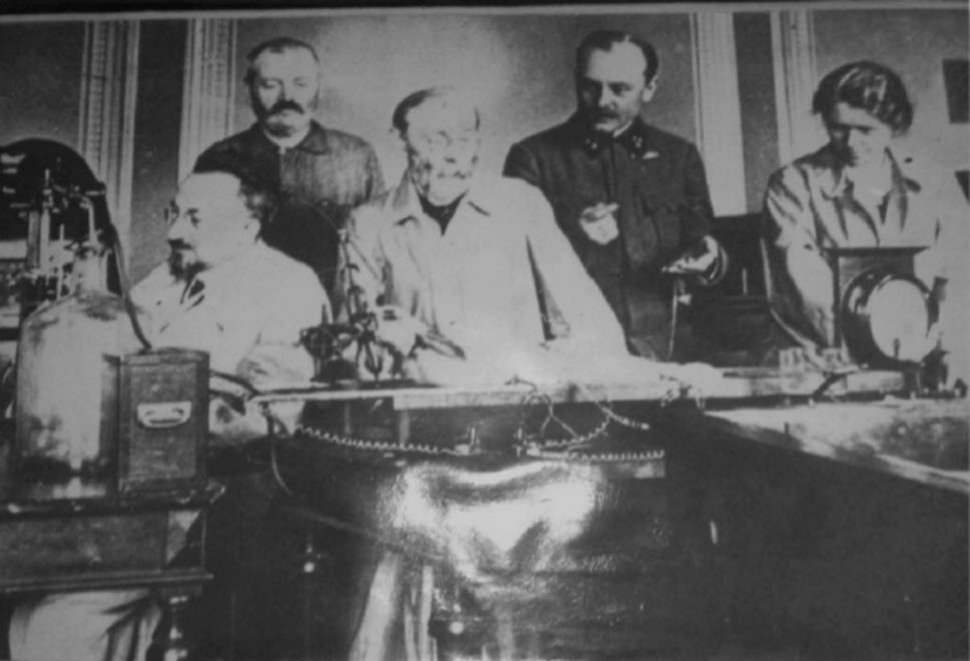
Gustaw Bikeles (front row, right) with Adolf Beck (front row, left) and co-workers during a neuro-electrophysiological experiment in the Department of Physiology of Lviv University. (Photograph from the collection of Danylo Halytsky Lviv National Medical University)

Gustaw Bikeles (Fig. 1) was born on September 1, 1861 into a Jewish family in Lemberg (then part of Austria–Hungary, today Lviv in Ukraine) [1]. After graduating from high school in his hometown, Bikeles began to study philosophy in Berlin, but he soon quit his studies and moved to Vienna, where he studied medicine. After his graduation in 1890, Bikeles came into contact with two eminent members the Viennese faculty at the time, Richard von Krafft-Ebing and Heinrich Obersteiner, and began his scientific activity under their supervision [1].

However, after some time in the 1890s, Bikeles returned to his home city of Lviv, where he worked in the Medical Faculty of Jan Kazimierz University and also ran a private neurological practice [1]. Bikeles initially worked at the University Department of Pathological Anatomy and the Clinic of Internal Medicine, and next in the Department of Physiology and the Department of Neurology [1]. Bikeles became a Dozent (lecturer) in 1900, was appointed associate professor in 1906, and promoted to full professor in 1913 [1]. During World War I, when the Russian army occupied Lviv, Bikeles escaped to Vienna, where he worked as a neurologist. After Russia's defeat, he returned to his hometown and continued his work at the University despite the war and a deteriorating health—at that time he almost lost his hearing completely. Bikeles died tragically on November 4, 1918, when he was hit in the head by a stray bullet during the battle of Lviv between the Ukrainian and Polish armies [1].

At the beginning of his scientific career in Vienna, Bikeles studied, among other themes, changes in the central nervous system after concussion. He was one of the first investigators to observe that concussion may result in foci of encephalomalacia and neurodegeneration in the brain and spinal cord in locations far away from the original injury [2]. Moreover, Bikeles noted that such changes primarily involved the myelin sheath, where they appeared earlier and more extensively than axons [2]. That work also became important in the discussion about the organic and functional pathogenetic mechanisms of post-traumatic neuroses [1]. Further, Bikeles addressed multiple sclerosis and was one of the early neurologists who observed inflammatory changes in the central nervous system during the course of this disease [3].

In Lviv, Bikeles mainly dealt with experimental neurophysiology and conducted experiments on dogs, cats, and rabbits. For many years, he was one of the closest collaborators and friends of Adolf Beck—a neurologist, pioneer of electroencephalography, and co-discoverer of bioelectrical potentials in the brain [1]. Together, they conducted electrophysiological experiments involving the stimulation of different parts of the nervous system with electrical or thermal stimuli and recording bioelectrical potentials (later known as evoked potentials) in the central nervous system. They used that method to study, among other issues, the interrelationships between the cerebral and the cerebellar cortex (demonstrating the influence of one cerebral hemisphere on the whole cerebellum) [4], the sensory functions of the cerebellum (showing bioelectrical potentials on the whole cerebellum after the stimulation of nerves regardless of their location) [5], the excitability of the cerebellar cortex [6] and various types of reflexes (showing that some reflexes are present after the damage of the cerebral cortex and/or cutting of the spinal cord and disproving the theory of the German neurologist Hermann Munk that the organization of afferent nerves entering the spinal cord is different for each limb joint) [7].

Moreover, Bikeles studied the distribution of sensory tracts in the spinal cord, analyzing reflexive regulation of blood pressure and other reflexes in physiological conditions and after injury to various parts of the central and peripheral nervous system, as well as describing the crossed pathways of the axons of sensory neurons in the spinal cord [8]. Another scientific topic that interested Bikeles was trophic neuronal function. He found that trophic changes do not only depend on cutting a nerve or excision of ganglia, and tactile corpuscles do not show a trophic character, but also the resumption of nerve function after injury may be connected with mechanisms not related to the nervous tissue per se [9].

Bikeles also published clinical works. For the first time, he described a sign that occurs when the patient outstretches the arm upward and backward with the elbow fully flexed. Extending the elbow causes resistance and pain during meningitis or brachial plexus neuritis. Such a sign is eponymously known as the Bikeles sign [10]. Moreover, Bikeles studied epilepsy (he tried to establish an animal model), as well the relationship of sweat secretion to neurological diseases [1].

Co-workers valued Bikeles for his diligence, inquisitiveness, and love of science. Eufemiusz Herman, a Polish neurologist and student of Bikeles wrote, “Gustaw Bikeles was the personification of a scientist with extraordinary diligence and a modest attitude, for whom life began and ended with science” [1].
